# DC-Link Voltage and Catenary Current Sensors Fault Reconstruction for Railway Traction Drives

**DOI:** 10.3390/s18071998

**Published:** 2018-06-22

**Authors:** Fernando Garramiola, Javier Poza, Jon del Olmo, Patxi Madina, Gaizka Almandoz

**Affiliations:** Faculty of Engineering, Mondragon Unibertsitatea, 20500 Arrasate-Mondragón, Spain; jpoza@mondragon.edu (J.P.); jdelolmo@mondragon.edu (J.d.O.); pmadina@mondragon.edu (P.M.); galmandoz@mondragon.edu (G.A.)

**Keywords:** sensor fault diagnosis, fault detection and isolation, fault injection, railway traction drive, sliding mode observer

## Abstract

Due to the importance of sensors in control strategy and safety, early detection of faults in sensors has become a key point to improve the availability of railway traction drives. The presented sensor fault reconstruction is based on sliding mode observers and equivalent injection signals, and it allows detecting defective sensors and isolating faults. Moreover, the severity of faults is provided. The proposed on-board fault reconstruction has been validated in a hardware-in-the-loop platform, composed of a real-time simulator and a commercial traction control unit for a tram. Low computational resources, robustness to measurement noise, and easiness to tune are the main requirements for industrial acceptance. As railway applications are not safety-critical systems, compared to aerospace applications, a fault evaluation procedure is proposed, since there is enough time to perform diagnostic tasks. This procedure analyses the fault reconstruction in the steady state, delaying the decision-making in some seconds, but minimising false detections.

## 1. Introduction

The availability of railway traction units can be improved by implementing condition-based maintenance (CBM) [1]. Fault diagnosis is needed in order to detect faults and implement such a maintenance strategy.

Several studies have been developed for the purpose of early fault detection. Mainly, diagnostic approaches are classified as model-based, signal-based and data-driven methods. A review of model-based and signal-based approaches is presented in ref. [2], whereas data-driven approaches are summarised in ref. [3]. Among signal-based methods, motor current signature analysis (MCSA) is a usual solution to detect faults in electric machines [4]. Data-driven methods [5,6,7,8] require a large amount of historic data, which demands high on-board data storage capacity [9] in moving systems, such as a train, due to the lack of high-frequency communication for remote diagnosis. On the other hand, model-based methods [10,11,12,13] require developing a model with regard to the knowledge of the system. Hybrid approaches, using model-based and data-driven methods, have been proposed in refs. [14,15].

In this research, an on-board model-based sensor fault diagnosis is proposed and implemented in a commercial traction control unit (TCU) for a railway application. In complex systems, a fault can concern several signals and, being a model-based fault diagnosis [16], a suitable solution to improve detection sensitivity [17]. Moreover, the traction control strategy requires sensor measurements, so a faulty sensor can result in a loss of availability and performance deterioration [18].

This research is focused on the DC-link voltage and catenary current sensors. The solution proposed is based on the model of the input filter for a railway traction drive. This model is simpler than the traction motor model and it has lower parameter variation during operation [19], thus, the implementation is easier and it does not require important computational resources. Similar to the model of the input filter, previous studies have used the model of a PWM rectifier [20,21] for DC-link voltage and catenary current sensor fault diagnosis. These studies have implemented the sensor fault diagnosis in test benches, but without any commercial railway TCU.

In this article, the solution proposed for sensor fault diagnosis is based on a sliding mode observer (SMO). SMO was initially introduced in refs. [22,23] as a robust control solution to model errors and measurement disturbances. In ref. [24], the theoretical background of SMO is explained. Based on the sliding mode control (SMC) concept, the SMC aims to lead a chosen variable into a sliding surface, and then maintain on it by means of a switching control. The first phase is called reaching mode and the second one is called sliding motion. The main advantage of SMO is the robustness under parameter uncertainties and measurement noises, whereas the main drawback is the chattering generated in the sliding motion. The SMO feedback gain is discontinuous, composed of a constant feedback gain matrix and a discontinuous vector [25], called the discontinuous injection signal. The discontinuous injection signal normally includes a gain constant and a discontinuous function, such as a sign, relay, or saturation. Later, smooth functions of the signal, such as a sigmoid function, have been used for online implementation in order to attenuate chattering [26]. In cases of discontinuous term elimination, the resulting observer is a Luenberger observer [27]. A Luenberger observer is the most popular observer for linear systems and deterministic settings, where the feedback gain matrix should be designed in order to correct the difference between measured and estimated outputs. A simple Luenberger observer with a relay or saturation function, can provide robustness against uncertainties or disturbances [28]. In ref. [25], a SMO with a discontinuous gain and an additional Luenberger-type gain matrix is proposed, in order to increase robustness. In ref. [29], a review of SMOs is presented, where several applications are mentioned. Recently, fault detection and isolation (FDI) [30,31] and sensorless control [32] are the main applications in electric drives.

In this article, a sensor fault reconstruction based on a SMO is proposed. One additional advantage of SMO, in comparison to other observers, is that it is possible to reconstruct the faults based on the equivalent control [33], called the equivalent injection signal, which represents the average value to maintain the sliding motion. The equivalent injection signal can be obtained by low-pass filtering or by a continuous approximation of the discontinuous injection signal. Once a fault occurs, if discontinuous injection signal is scaled to the estimation error to detect, and the equivalent injection signal value will change to maintain the sliding motion. In the case of abrupt faults over the maximum fault to detect, the sliding motion is destroyed.

Recently, several publications propose a SMO for sensor FDI. In ref. [21], SMO-based FDI approaches for DC-link voltage and catenary current sensors are presented. Residuals are obtained from the difference between measured and estimated values, and they are compared to thresholds for decision-making. Neither severity of fault nor fault reconstruction is estimated. In ref. [30], a FDI for the DC-link voltage is proposed, but depending on the motor model, it has a more complex and, consequently, a higher parameter variability during operation than the input filter model proposed here. Moreover, fault reconstruction is not proposed. In ref. [34], SMO-based approaches are used for FDI in phase current and rotor position sensors in a permanent magnet synchronous generator (PMSG) for a wind turbine application. In refs. [35,36], a SMO for phase current sensor fault reconstruction in permanent magnet synchronous machines (PMSM) is presented. An augmented system is presented in order to define sensor faults as actuator faults. Thus, sensor fault reconstruction is obtained based on the equivalent injection signal [37]. Fault reconstruction is validated in a hardware-in-the-loop platform without a commercial TCU.

In contrast to previous publications, in this article, a fault reconstruction based on a SMO for DC-link voltage and catenary current sensor faults is proposed. Previous studies applied to DC-links are limited to FDI without fault reconstruction. Moreover, the solution is validated in a hardware-in-the-loop (HIL) platform, composed of a real-time simulator and a commercial TCU for a railway application. The TCU is a commercial unit for a tram, developed by CAF Power and Automation. This study analyses the main fault modes in sensors: offset and gain faults. An early fault diagnosis is implemented in order to avoid a failure, increasing the availability and reliability of the traction system. The solution proposed provides both the FDI and severity of the fault. An easy to tune solution is proposed in the face of input filter parameter variations and fault magnitude to be detected. Thus, the proposed solution is simple to adapt to other railway traction drive configurations.

The paper has the following structure: Section 2 presents the railway traction drive description and problem statement. Section 3 proposes a SMO for DC-link voltage and catenary current sensors. Section 4 proposes a fault diagnosis and reconstruction approach for DC-link voltage and catenary current sensors. In Section 5, the validation in a HIL platform is presented. Finally, the discussion and conclusions are given.

## 2. Railway Traction Unit Description and Input Filter Model

**Figure 1:**
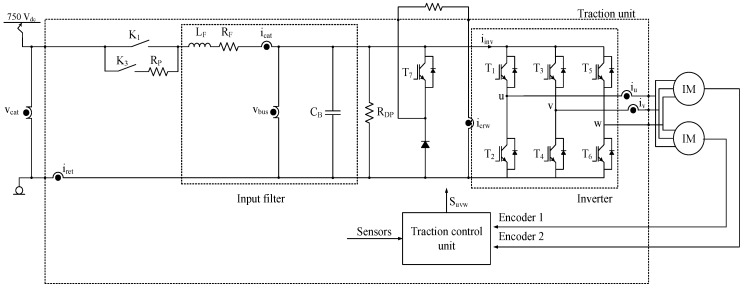
Railway traction unit.

There are different traction unit topologies, but this research is based on the input filter of the traction unit shown in Figure 1. The sensors in the traction unit are summarized in Table 1.

**Figure 2:**
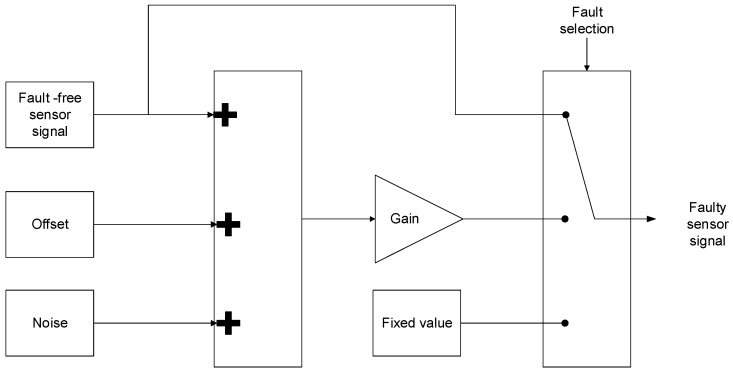
Sensor fault injection.

Similar to previous publications [38,39], the model of the input filter in state space is presented in Equation (1), being $x^{T}=\left[\begin{array}{cc} i_{cat} & v_{bus} \end{array}\right]$, $u^{T}=\left[\begin{array}{ccc} v_{cat} & i_{inv} & i_{crw} \end{array}\right]$ and $y^{T}=\left[\begin{array}{cc} i_{cat} & v_{bus} \end{array}\right]$. $i_{inv}$ is not directly measured, it is calculated from T1, T3, T5 switch states and $i_{u}$ and $i_{v}$ current sensor measurements. The sensor faults are represented as ${\left[\begin{array}{cc} f_{icat} & f_{vbus} \end{array}\right]}^{T}$. Different fault modes and noise can be injected, as is shown in Figure 2.

(1) $$\frac{dx}{\mathrm{dt}}=\left[\begin{array}{cc} -\frac{R_{F}}{L_{F}} & -\frac{1}{L_{F}}\\ \frac{1}{C_{B}} & 0 \end{array}\right]x+\left[\begin{array}{ccc} \frac{1}{L_{F}} & 0 & 0\\ 0 & -\frac{1}{C_{B}} & -\frac{1}{C_{B}} \end{array}\right]u\;y=\left[\begin{array}{cc} 1 & 0\\ 0 & 1 \end{array}\right]x+\left[\begin{array}{c} f_{icat}\\ f_{vbus} \end{array}\right].$$

A new state z, which is a filtered version of y, and given by Equation (2), is proposed. Being $z^{T}=\left[\begin{array}{cc} i_{cat\_f} & v_{bus\_f} \end{array}\right]$ and $y^{T}=\left[\begin{array}{cc} i_{cat} & v_{bus} \end{array}\right]$. $A_{f}=\left[\begin{array}{cc} A_{f1} & 0\\ 0 & A_{f2} \end{array}\right]$ is a positive definite diagonal matrix that represents inverse time constants [40]:

(2) $$\dot{z}=-A_{f}z+A_{f}y$$

Thus, an augmented system is represented in (3). Sensor faults are analysed as actuator faults in the augmented system:

(3) $$\left[\begin{array}{c} \dot{x}\\ \dot{z} \end{array}\right]=\underset{A_{0}}{\underbrace{\left[\begin{array}{cccc} -\frac{R_{F}}{L_{F}} & -\frac{1}{L_{F}} & 0 & 0\\ \frac{1}{C_{B}} & 0 & 0 & 0\\ A_{f1} & 0 & -A_{f1} & 0\\ 0 & A_{f2} & 0 & -A_{f2} \end{array}\right]}}\left[\begin{array}{c} x\\ z \end{array}\right]+\underset{B_{0}}{\underbrace{\left[\begin{array}{ccc} \frac{1}{L_{F}} & 0 & 0\\ 0 & -\frac{1}{C_{B}} & -\frac{1}{C_{B}}\\ 0 & 0 & 0\\ 0 & 0 & 0 \end{array}\right]}}u+\left[\begin{array}{cc} 0 & 0\\ 0 & 0\\ A_{f1} & 0\\ 0 & A_{f2} \end{array}\right]\left[\begin{array}{c} f_{icat}\\ f_{vbus} \end{array}\right]\;z=\underset{C_{0}}{\underbrace{\left[\begin{array}{cccc} 0 & 0 & 1 & 0\\ 0 & 0 & 0 & 1 \end{array}\right]}}\left[\begin{array}{c} x\\ z \end{array}\right].$$

Observability and controllability of the augmented system represented in Equation (3) is checked. The controllability for a linear system is given if expression Equation (4) is fulfilled, n being the dimension of the state vector ${\left[x\;z\right]}^{T}$. The rank obtained is 4, so it can be concluded that the system is fully controllable:

(4) $$rank\left(B_{0}\vdots A_{0}B_{0}\right)=n$$

The next step is to check the observability of the system, given if Equation (5) is fulfilled. The rank is 4, so it can be concluded that the system is fully observable:

(5) $$rank\left(\begin{array}{c} C_{0}\\ \cdots \\ A_{0}C_{0} \end{array}\right)=n$$

## 3. Sliding Mode Observer for DC-Link Voltage and Catenary Current Sensors

**Figure 3:**
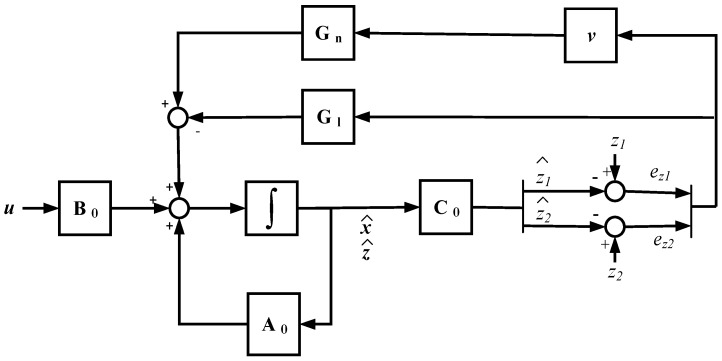
Sliding mode observer structure.

The proposed SMO is presented in Figure 3. The SMO has two feedback gains, a linear gain given by the matrix **G_l_**, and a nonlinear gain, composed of a matrix **G_n_** and discontinuous term *v*. The addition of the linear gain matrix **G_l_** provides robustness against uncertainties in order to be stable [25]. The discontinuous term is given by Equation (6), where p is a gain that should be higher than the maximum sum of sensor faults and uncertainties, in order to achieve the sliding motion, sat is the bound of the sliding surface and $e_{z}=\left[e_{z1}e_{z2}\right]=\hat{z}-z$, $\hat{z}$ being the estimated states and z the filtered measurements given by Equation (2).

(6) vi={−pAf(1sat)eziif ezi>sat or ezi<−sat−pAf(ezi|ezi|)if sat≥ezi≥−sat

Observer equations are given in Equation (7). The proposed gain matrices, as is shown in ref. [41], are presented in Equation (8). $-A_{f1}$ and $-A_{f2}$ correspond to the additional observer open loop poles for the new augmented system, apart from the original system poles, whereas −Χ sets the additional closed loop poles, as it can be seen in Equations (7) and (9), respectively. $A_{f1}$, $A_{f2}$, and Χ are positive constants, where Χ is higher than $A_{f1}$ and $A_{f2}$.

(7) $$\left[\begin{array}{c} \dot{\hat{x}}\\ \dot{\hat{z}} \end{array}\right]=\underset{A_{0}}{\underbrace{\left[\begin{array}{cccc} -\frac{R_{F}}{L_{F}} & -\frac{1}{L_{F}} & 0 & 0\\ \frac{1}{C_{B}} & 0 & 0 & 0\\ A_{f1} & 0 & -A_{f1} & 0\\ 0 & A_{f2} & 0 & -A_{f2} \end{array}\right]}}\left[\begin{array}{c} \hat{x}\\ \hat{z} \end{array}\right]+\underset{B_{0}}{\underbrace{\left[\begin{array}{ccc} \frac{1}{L_{F}} & 0 & 0\\ 0 & -\frac{1}{C_{B}} & -\frac{1}{C_{B}}\\ 0 & 0 & 0\\ 0 & 0 & 0 \end{array}\right]}}u-G_{l}\left[\begin{array}{c} e_{z1}\\ e_{z2} \end{array}\right]G_{n}\left[\begin{array}{c} v_{1}\\ v_{2} \end{array}\right]\;\hat{z}=\underset{C_{0}}{\underbrace{\left[\begin{array}{cccc} 0 & 0 & 1 & 0\\ 0 & 0 & 0 & 1 \end{array}\right]}}\left[\begin{array}{c} \hat{x}\\ \hat{z} \end{array}\right].$$

(8) $$G_{l}=\left[\begin{array}{cc} 0 & 0\\ 0 & 0\\ 1 & 0\\ 0 & 1 \end{array}\right],\;G_{n}=\left[\begin{array}{cc} 0 & 0\\ 0 & 0\\ -A_{f1}+Χ & 0\\ 0 & -A_{f2}+Χ \end{array}\right].$$

The system error given in Equation (9) is obtained from the difference between Equations (3) and (7). The estimated states $\hat{x}$ are not influenced by feedback gains, so they are open loop estimations, based on the observer model and input vector $u^{T}=\left[\begin{array}{ccc} v_{\mathrm{cat}} & i_{\mathrm{inv}} & i_{\mathrm{crw}} \end{array}\right]$. Thus, these estimations do not depend on $i_{\mathrm{cat}}$ and $v_{bus}$ sensor measurements and, consequently, an error arises in $e_{x1}$ or $e_{x2}$ in the case of a faulty $i_{\mathrm{cat}}$ or $v_{bus}$ sensor, respectively. On the other hand, estimated states $\hat{z}$ are closed loop estimations, so the errors $e_{z1}$ and $e_{z2}$ are reduced due to feedback gains, but this generates a change in the nonlinear gain average value, which allows the fault reconstruction.

(9) $$\left[\begin{array}{c} \dot{e_{x}}\\ \dot{e_{z}} \end{array}\right]=\left[\begin{array}{cccc} -\frac{R_{F}}{L_{F}} & -\frac{1}{L_{F}} & 0 & 0\\ \frac{1}{C_{B}} & 0 & 0 & 0\\ A_{f1} & 0 & -Χ & 0\\ 0 & A_{f2} & 0 & -Χ \end{array}\right]\left[\begin{array}{c} e_{x}\\ e_{z} \end{array}\right]+G_{n}\left[\begin{array}{c} v_{1}\\ v_{2} \end{array}\right]-\left[\begin{array}{cc} 0 & 0\\ 0 & 0\\ A_{f1} & 0\\ 0 & A_{f2} \end{array}\right]\left[\begin{array}{c} f_{icat}\\ f_{vbus} \end{array}\right].$$

The sliding mode surface is defined as $s=\{\left(e_{x},\;e_{z}\right)|e_{z}=0\}$, in order to force output error $e_{z}$ to zero and achieve sliding motion. The Lyapunov candidate $V=\frac{1}{2}e_{z}^{T}e_{z}=\frac{1}{2}\left(e_{z1}^{2}+e_{z2}^{2}\right)$ is chosen for stability analysis. Thus, Equation (10) should be fulfilled. Furthermore, we select a p that ensures $\dot{e_{z1}}e_{z1}<0$ and $\dot{e_{z2}}e_{z2}<0$. The procedure for $\dot{e_{z1}}e_{z1}$ is shown in Equation (11):

(10) $$\dot{V}=\dot{e_{z1}}e_{z1}+\dot{e_{z2}}e_{z2}<0$$

(11) $$\dot{e_{z1}}e_{z1}=\left(A_{f1}e_{x1}-{Χe}_{z1}-A_{f1}v_{1}+Χv_{1}-A_{f1}f_{icat}\right)e_{z1}\;=A_{f1}\left(e_{x1}-f_{icat}\right)e_{z1}-{Χe}_{z1}^{2}+p\left(A_{f1}^{2}\frac{e_{z1}}{\left|e_{z1}\right|}-ΧA_{f1}\frac{e_{z1}}{\left|e_{z1}\right|}\right)\;=A_{f1}\left(e_{x1}-f_{icat}\right)e_{z1}-{Χe}_{z1}^{2}+p\left|e_{z1}\right|\left(A_{f1}^{2}-ΧA_{f1}\right)<0$$

Assuming that $\left|e_{x1}\right|\leq \left|f_{icat}\right|+\left|d\right|$, d being the uncertainties in the system, and $Χ-A_{f1}>0$, p should fulfil Equation (12), for any faulty case:

(12) $$p>\frac{e_{z1}}{\left|e_{z1}\right|}\left(\frac{e_{x1}-f_{icat}}{Χ-A_{f1}}\right)-\frac{\left|e_{z1}\right|}{A_{f1}\left(Χ-A_{f1}\right)},$$

A simpler condition is obtained in Equation (13). As the second term is always positive, we can obtain an expression with a higher value, which only depends on the maximum fault to detect, bounded uncertainties, and user-defined parameters Χ and $A_{f1}$. The maximum fault to detect should be under the sensor safety level, otherwise protections are enacted. Thus, if p is over the value of that expression, the Lyapunov condition is fulfilled:

(13) $$\frac{e_{z1}}{\left|e_{z1}\right|}\left(\frac{e_{x1}-f_{icat}}{Χ-A_{f1}}\right)-\frac{\left|e_{z1}\right|}{A_{f1}\left(Χ-A_{f1}\right)}<\frac{e_{z1}}{\left|e_{z1}\right|}\left(\frac{\left|e_{x1}\right|+\left|f_{icat}\right|}{Χ-A_{f1}}\right)<\frac{2\left|f_{icat}\right|+\left|d\right|}{Χ-A_{f1}},\;p>\frac{2\left|f_{icat}\right|+\left|d\right|}{Χ-A_{f1}}.$$

Similarly, the same is obtained for $\dot{e_{z2}}e_{z2}$. Thus, p should fulfil the two conditions given in Equation (14). Both, sensor faults and uncertainties are assumed to be unknown but bounded:

(14) $$p>\frac{2{\left|f_{icat}\right|}_{max}+{\left|d\right|}_{max}}{Χ-A_{f1}},\;p>\frac{2{\left|f_{vbus}\right|}_{max}+{\left|d\right|}_{max}}{Χ-A_{f2}}.$$

Despite different methods presented in the literature for SMO design, as linear matrix inequality (LMI) [42,43], or adaptive SMO [44], which can avoid the overestimation of discontinuous gain and reduce chattering in the estimated variables, this article proposes a simple solution to design the observer, for railway application requirements. Low computational requirements and easiness to tune, in the case of input filter parameter variation and maximum fault magnitude to be detected, are the main factors for the observer design selection.

The sliding mode observer proposed in Equation (7) and shown in Figure 3 is implemented in MATLAB-Simulink. Different sensor fault modes are injected, additives as offset faults and multiplicatives as gain faults, as well as the measurement noise. Moreover, offset and gain faults are injected in several sensors at the same time. In all the cases, the DC-link voltage and catenary current sensor faults are reconstructed.

## 4. Fault Diagnosis and Reconstruction for DC-Link Voltage and Catenary Current Sensors

**Figure 4:**
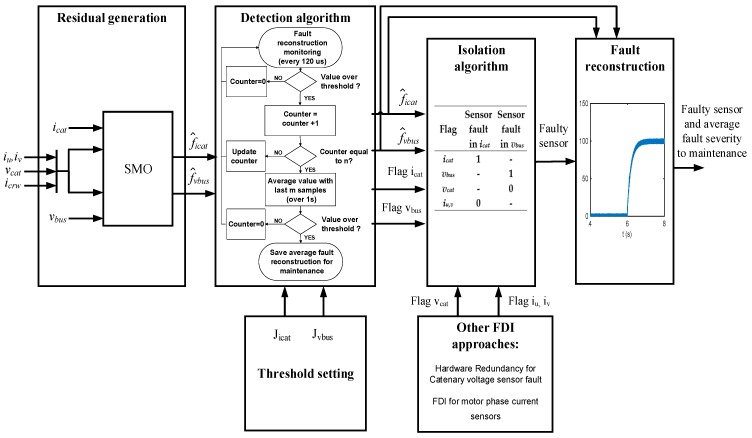
Fault diagnosis and reconstruction for DC-link voltage and catenary current sensors.

The fault diagnosis solution will be divided in the following steps: residual generation, threshold setting, fault detection, and isolation. The severity of the fault is given by the residual proposed, as it is a reconstruction of the fault. Thus, a complete fault diagnosis for the DC-link voltage and catenary sensors is being proposed here, and shown in Figure 4. There are some cases where additional FDI developed in a previous work [39] are recommended in order to isolate the faulty sensor.

### 4.1. Residual Generation

**Figure 5:**
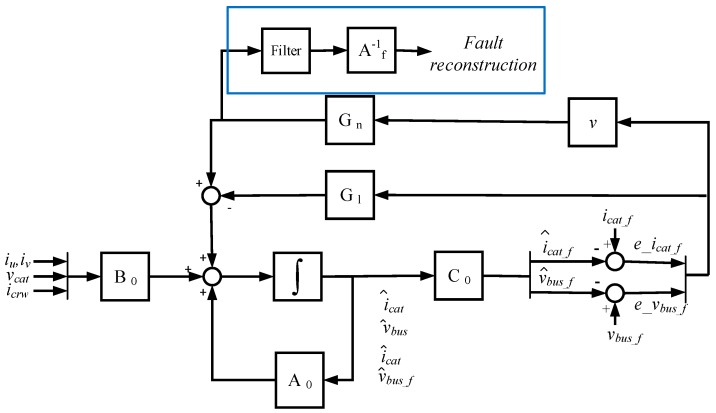
Sensor fault reconstruction for DC-link voltage and catenary current sensors.

Similar to [40,41], sensor fault reconstruction is given by ${\hat{f}}_{i}={\left(A_{f}{}_{i}\right)}^{-1}v_{i}{}_{eq}$, $v_{i}{}_{eq}$ being a filtered version of the $G_{n}$ matrix gain outputs, as is shown in Figure 5. Thus, the sensor fault reconstructions will be chosen as residuals, one being the residual for the catenary current sensor and another residual for the DC-link voltage. In the absence of differences between the real system and the model, the residuals should be zero for a fault-free case.

**Figure 6:**
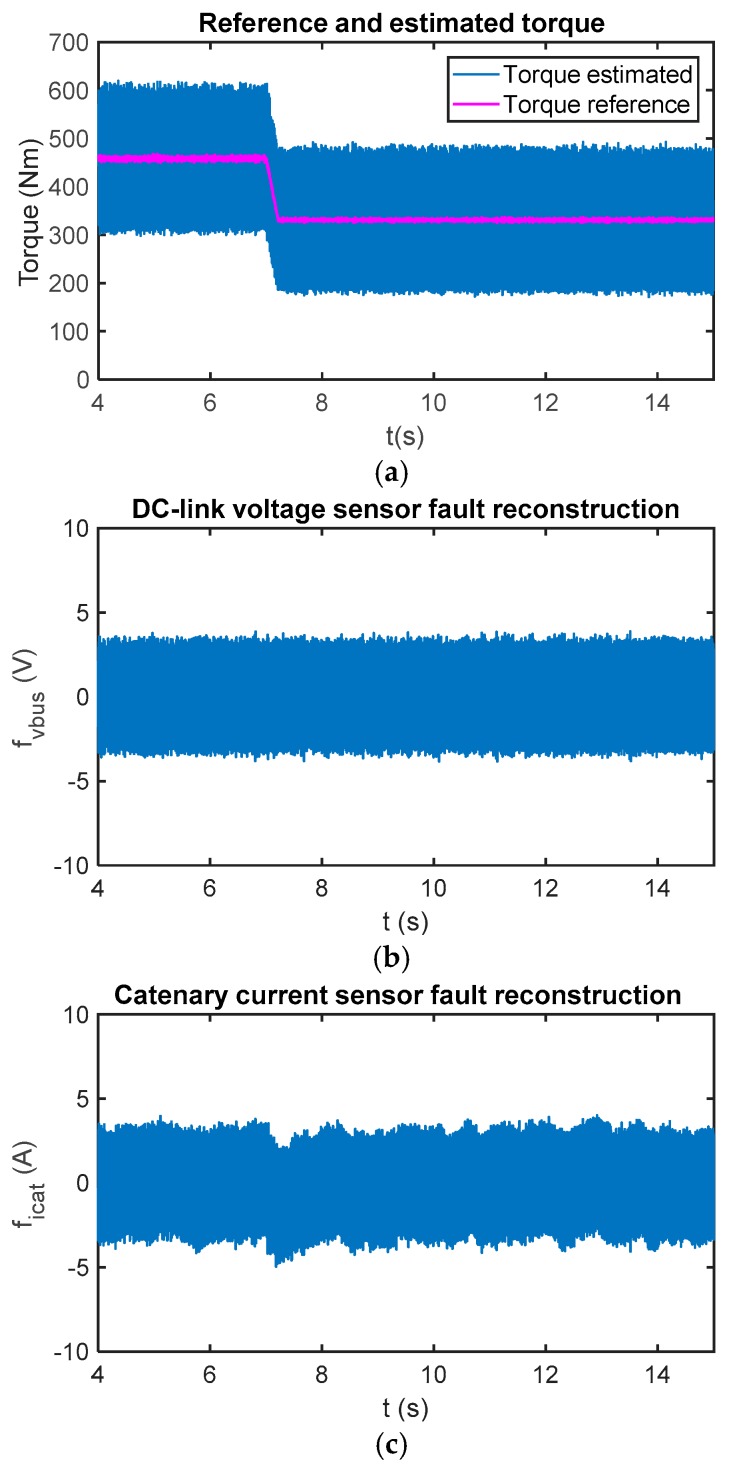
(**a**) Reference and estimated motor torque; (**b**) DC-link voltage sensor reconstruction in the case of a fault-free sensor; and (**c**) catenary current sensor reconstruction in the case of a fault-free sensor.

Due to uncertainties in the input model and in the iinv calculation, as it is obtained from the phase current measurements and power switch states, the reconstruction can be different from zero during the fault-free case. Furthermore, due to the sliding movement and chattering effect, an oscillation arises in the reconstruction. The chattering is caused by the injection signal and the executing period. The oscillation amplitude is related to the low-pass filter implemented to obtain the reconstruction, as well. A low cut-off frequency to allow passing fault dynamics, but to eliminate high frequency noises should be selected. A cut-off frequency equal to 5 Hz has been selected for this railway application. In Figure 6, reconstructions for the sensor fault-free case and a torque change are presented.

The dynamic response and robustness of the proposed observer for residual generation, under torque changes, catenary voltage changes and input filter parameter variation is analysed. Furthermore, a brief comparison to a Luenberger observer under measurement noise is made.

#### 4.1.1. Dynamic Response and Robustness under Torque and Catenary Voltage Changes

**Figure 7:**
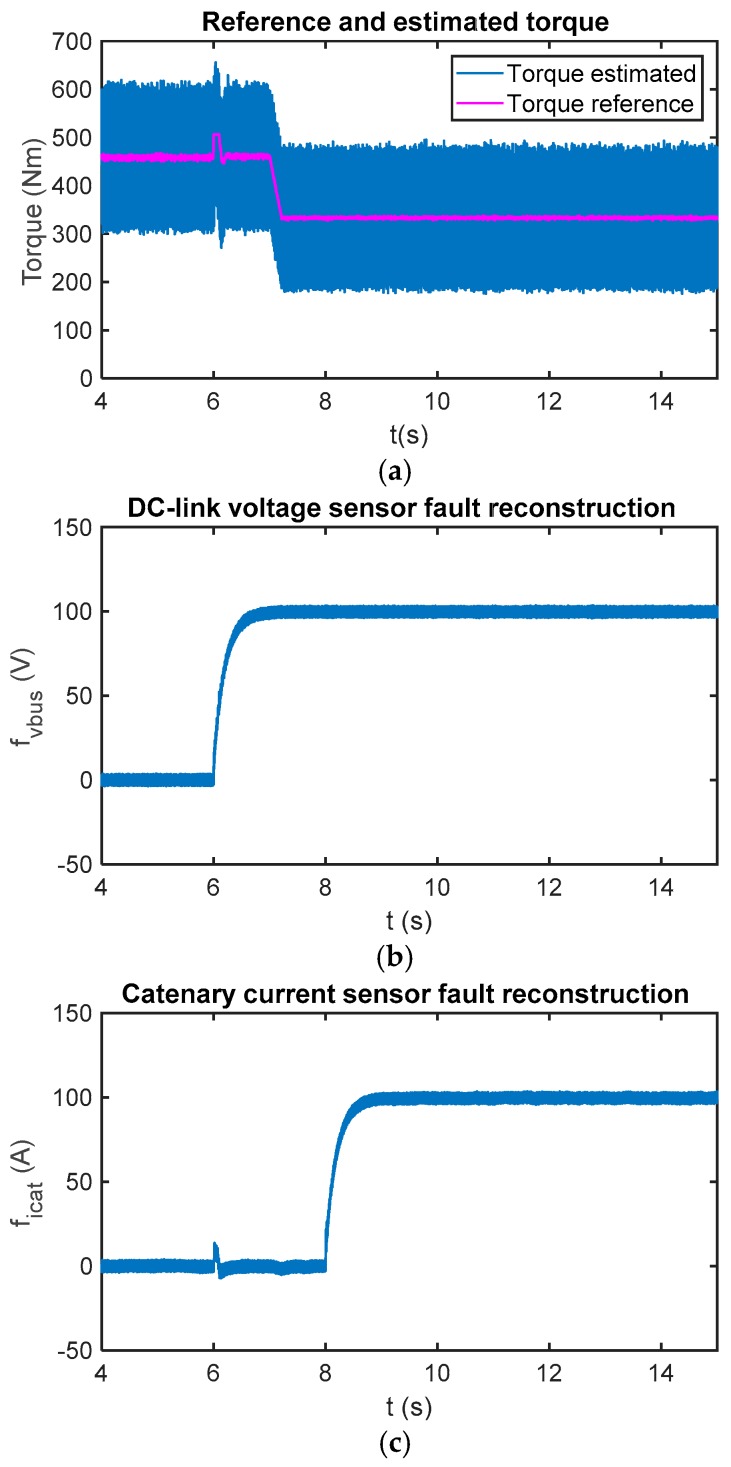
(**a**) Reference and estimated motor torque; (**b**) DC-link voltage sensor reconstruction in the case of a fault injected in the sensor at 6 s; and (**c**) catenary current sensor reconstruction in case of fault injected in the sensor at 8 s.

Offset faults are injected into the DC-link voltage and catenary current sensors. At *t* = 6 s, 100 V are injected into the DC-link voltage sensor, whereas a 100 A offset is injected into the catenary current sensor at *t* = 8 s. As can be seen in Figure 7, both reconstructions are decoupled in the steady state. Moreover, torque change does not result in a fault reconstruction change in the steady state.

The fault reconstruction dynamics are given by system closed loop poles, given by −Χ and the reconstruction filter frequency. As mentioned before, a low-pass filter with a cut-off frequency equal to 5 Hz has been chosen in order to reduce the oscillation. A railway traction unit is not a safety-critical system, as the control strategy can stop and restart the traction unit in seconds, maintaining the motion of the train. Thus, there is no need for an instantaneous detection, and decision-making is done after maintaining the reconstruction over the threshold for some seconds. Thus, a low frequency for filtering transients has been chosen in order to reduce the possibility of false detections.

**Figure 8:**
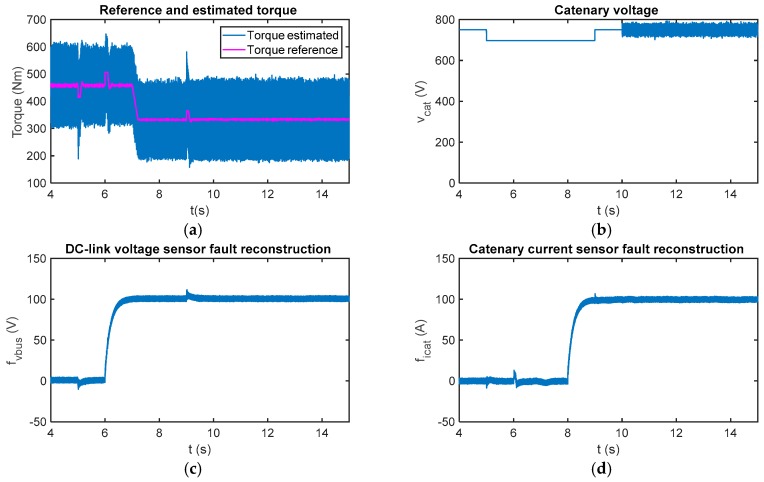
(**a**) Reference and estimated motor torque; (**b**) catenary voltage measurement; (**c**) DC-link voltage sensor reconstruction in the case of abrupt changes in the catenary voltage and measurement noise; and (**d**) catenary current sensor reconstruction in case of abrupt changes in catenary voltage and measurement noise.

The fault reconstruction will be analysed in the case of abrupt changes in the catenary voltage. In Figure 8, the fault reconstruction is presented for offset faults in the DC-link voltage and catenary current sensors, similar to previous simulations. Moreover, a catenary voltage drop from 750 V to 700 V is injected. During the catenary voltage decrease and increase, a transient arises in the DC-link voltage sensor fault reconstruction, which lasts around 0.5 s. The effect on the catenary current sensor fault reconstruction is negligible. Injected noise in the catenary voltage measurement, starting at *t* = 10 s, does not have an influence in the fault reconstructions.

#### 4.1.2. Sensitivity under Input Filter Parameter Variations

The fault reconstruction under input filter parameter variations have been analyse, as well. The parameters are the series resistance $R_{F}$ and inductance $L_{F}$, and the DC-link capacitor $C_{B}$. In ref. [21], it was proposed to analyse a variation of ±100% for line resistance and inductance for a railway application. Thus, in this article the sensibility of fault reconstructions for the variation of ±100% for two of the three aforementioned parameters will be analysed. In the case of capacitor variation, a variation of −100% does not make sense.

Similar to previous simulations, a 100 V offset will be injected in DC-link voltage sensor at *t* = 6 s and 100 A offset in catenary current sensor at *t* = 8 s. The average value of both reconstructions will be calculated in the period from *t* = 13 s to *t* = 15 s.

**Figure 9:**
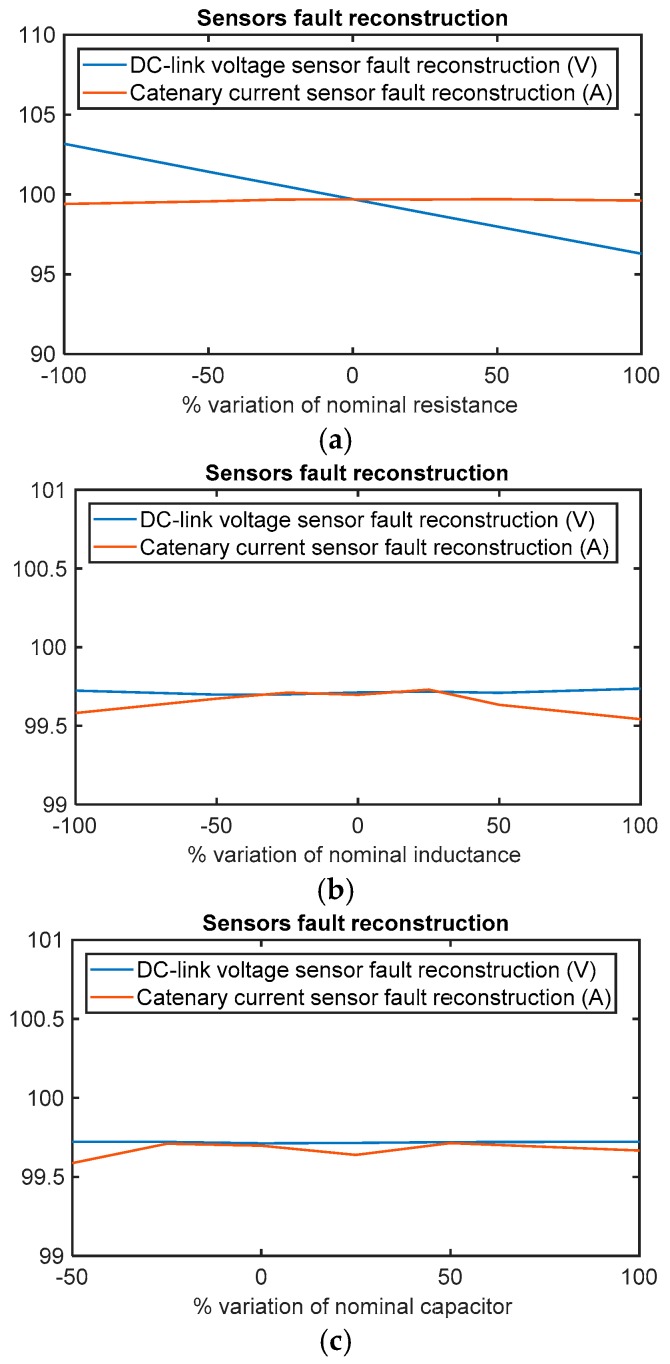
Sensor fault reconstruction deviation for a motor reference torque of 332 Nm under +100 V offset fault in the DC-link sensor and +100 A offset fault in the catenary current sensor. (**a**) Sensors fault reconstruction deviation for resistor variation; (**b**) sensor fault reconstruction deviation for inductance variation; and (**c**) sensors fault reconstruction deviation for capacitor variation.

As it is shown in Figure 9, the fault reconstruction is robust to parameter variation, the DC-link voltage sensor fault reconstruction under resistance variations being the most sensitive. The deviation from injected value in the DC-link voltage sensor reconstruction is proportional to the variation of the resistor and the catenary current. Thus, the deviation in the DC-link voltage sensor fault reconstruction increases for higher torque references. The maximum deviation is given for a maximum motor torque reference of 690 Nm and a resistance value equal to twice the nominal value, the deviation being 7.88 V. In any case, the deviation between the fault reconstruction and the injected fault is under 10 V.

Thus, although high parameter variations have been analysed, the fault reconstruction deviation is not relevant for system performance. The highest deviation occurs in the DC-link voltage sensor fault reconstruction due to the series resistor variation. The simplicity of input filter model for the observer design, with only the three aforementioned parameters, is an important advantage to make the sensitivity analysis easier, compared to models that utilise the motor model, where several parameters can change during motor operation.

#### 4.1.3. Brief Comparison of SMO and Luenberger Observer under Measurement Noise

**Figure 10:**
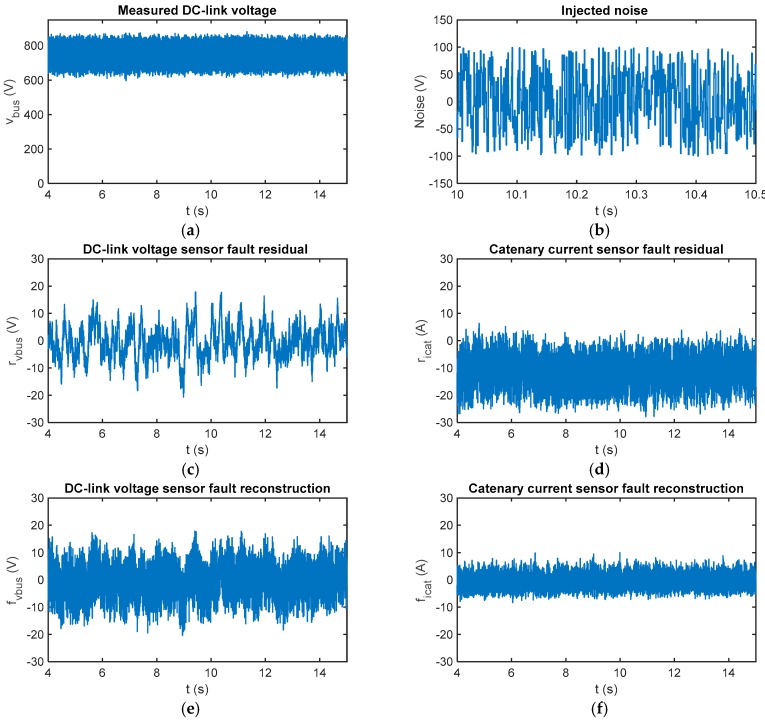
A comparison of Luenberger and sliding mode observer-based fault estimations under noisy measured DC-link voltage and sensor fault-free cases. (**a**) Measured DC-link voltage with noise injected; (**b**) injected noise signal; (**c**) DC-link voltage sensor fault estimation with a Luenberger observer; (**d**) catenary current sensor fault estimation with a Luenberger observer; (**e**) DC-link voltage sensor fault estimation with SMO; and (**f**) catenary current sensor fault estimation with SMO.

In Figure 10a comparison of sensitivity between a Luenberger observer, presented in ref. [39], and the proposed SMO, under measurement noises for a sensor fault-free case is presented. A random signal with uniform distribution is injected in the DC-link voltage measurement. Thus, the fault reconstructions based on SMO are robust, being the average value near zero. The SMO based residuals are not influenced by the measurement noise injection. On the other hand, the catenary current residual based on the Luenberger observer is influenced by the measurement noise, increasing the average residual up to –10.78 A for a fault free case. The DC-link voltage sensor fault based on Luenberger observer is not significantly affected. Thus, SMO-based residuals show a better response under measurement noises in the sensors.

In our application, as a quick detection is not needed, the robustness of the fault estimation to measurement noise is an important advantage in relation to previously-developed Luenberger observer-based FDI.

### 4.2. Threshold Setting

**Figure 11:**
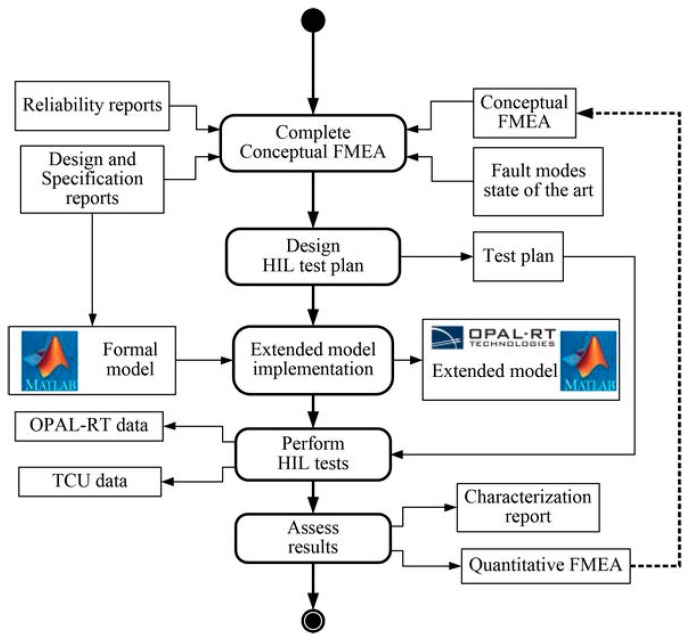
Flowchart for system analysis under the fault methodology.

The thresholds for both residuals have been set based on traction drive analysis under faults. The minimum fault to detect has been defined based on quantitative effects of sensor faults in the traction system. The methodology to obtain the quantitative effects was presented in ref. [45], and summarized in Figure 11. System analysis for sensor fault-free cases has been don, as well, generating the residuals in the case of input filter parameter variations and noise injection in sensor measurements. Moreover, the residuals have been obtained for different operating points in order to identify the residual sensitivity.

The key point is to avoid any false alarm. For that, a norm-based residual evaluation has been chosen, the peak norm. Thus, the threshold should be set over the peak value of residuals obtained for any sensor fault-free cases. After traction system analysis, the threshold for the DC-link voltage sensor has been set to ±20 V, whereas the threshold for the catenary current has been set to ±20 A. Sensor faults under these thresholds are missed, but they are not relevant as the deviation in torque estimation is under 2%, and in power returned to the catenary is under 5%.

### 4.3. Fault Detection and Isolation

**Figure 12:**
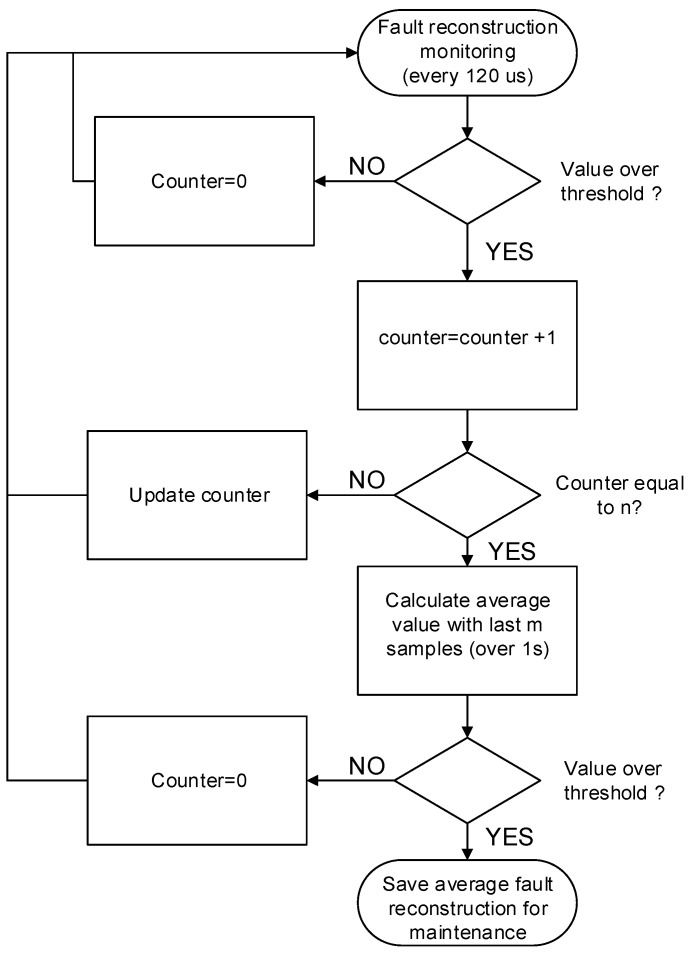
Flowchart for fault detection evaluation.

As previously mentioned, as a train is not a safety-critical system, the control can stop and restart the traction unit during some seconds, maintaining the train in motion. Thus, an instantaneous fault detection is not compulsory, although the algorithm could do it in a short time period lower than 1 s, for the observer parameter chosen in the proposed solution. Hence, an evaluation procedure for fault detection is proposed in order to increase the robustness of the evaluation and minimize false detections. The evaluation flowchart for each sensor fault is presented in Figure 12, once the fault reconstruction surpasses the prefixed threshold, a counter is activated. If reconstruction remains above the threshold during n samples, a flag is activated and the following m samples are stored. If the average value of m samples is over the threshold, a sensor fault decision is made. The sensor fault and the reconstruction average value are transferred to a maintenance expert. The counter value n and the number of samples m have been chosen so that the procedure from fault to decision-making lasts some seconds. A reasonable time period for this railway application is around 10 s.

This research work is focused in the FDI for DC-link voltage and catenary current sensors. Two residuals have been presented, which allow the isolation of the DC-link voltage and catenary current sensor faults. On the other hand, it assumes that the motor phase sensors (*i_u,v_*), catenary voltage sensor (*v_cat_*), and braking unit current sensor (*i_crw_*), which are inputs of the input filter system presented, are fault-free or detected with other FDI approaches. The logic and FDI algorithms for this sensor was presented in ref. [39]. A summary of those approaches is presented here. In the case of the catenary voltage sensor, the available sensor redundancy in other traction units should be checked in order to distinguish DC-link voltage and catenary voltage sensor faults. In the case of motor phase sensor faults, the solution proposed here is robust to offset faults in the steady state, allowing to distinguish between phase sensor and catenary current sensor faults. However, in the case of high gain faults, due to the error calculating current park components (*i_d_* and *i_q_*) in the control strategy, the strategy can lead to the wrong conclusion that there is a fault in the catenary current sensor. Thus, the FDI method proposed for the phase current sensor in the aforementioned article [39] should be checked. This approach is based on filtering the current park components, due to the oscillation that arises in these current components in the case of a phase current sensor fault. The frequency of the oscillation is equal to the fundamental frequency of the motor stator current for offset faults, and twice that for gain faults. In the case of the braking unit current sensor, it does not have any effect during traction, so in the case of a faulty *i_crw_* sensor, it could be detected during braking.

In Table 2 the logic to isolate faults in DC-link voltage and catenary current sensors is summarized. If the flag is equal to 1, it indicates that the flag should be activated, and if it is equal to 0, it means that the flag is not activated, whereas “-“ means that it does not mind the state of the flag. This logic shows that it is possible to isolate faults in DC-link voltage and catenary current sensors at the same time. As a drawback, there are two cases to keep in mind in order to avoid false alarms related to the flags being equal to 0 in Table 2. First, in the case of the catenary current sensor, it is recommended to apply a FDI to the motor phase current sensors to avoid a false evaluation in the catenary current sensor due to the effect in the control strategy of a high gain fault in any motor phase current sensor. In case of the DC-link voltage, the available hardware redundancy for the catenary voltage sensor should be checked in order to avoid a false evaluation in the DC-link voltage sensor, as the same fault in both sensors has a similar residual, with the same amplitude, but opposite sign.

## 5. Hardware-In-The-Loop Validation of Fault Reconstruction

**Figure 13:**
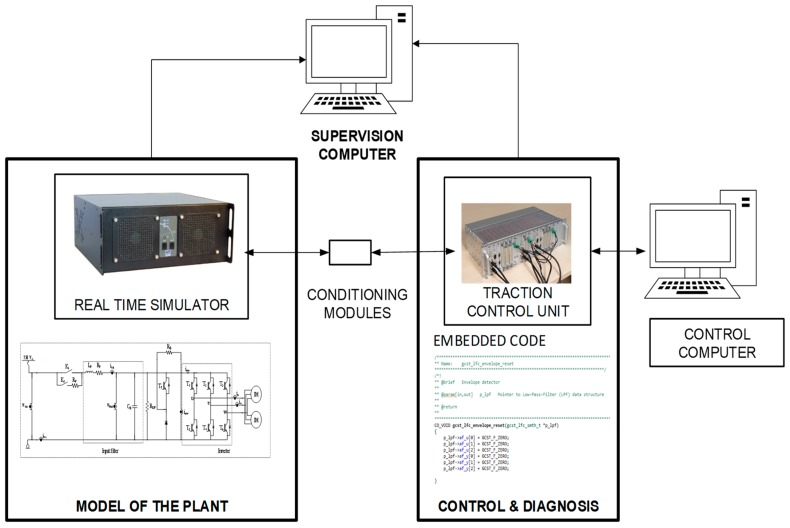
Hardware-in-the-loop platform.

The results presented in the previous section have been validated in a HIL platform, shown in Figure 13. The platform is composed of a real-time simulator, where the railway traction unit is modelled in MATLAB-Simulink, and a commercial TCU, developed by CAF Power and Automation, for a railway application, where the proposed observer and reconstruction embedded code is implemented.

The TCU is externally connected to the real-time simulator through analogue and digital ports. The fault diagnosis algorithms and the control strategy for the traction unit are running on the TCU. Conditioning modules to adapt the inputs and outputs between the TCU and the real-time simulator are needed.

This platform allows injecting faults, easily and quickly, in order to test the different FDI approaches. The simulation step for model running in the real-time simulator is 15 µs. The TCU has a DSP for high-speed execution. The sensor measurements are captured and monitored every 120 µs for validation purposes.

**Figure 14:**
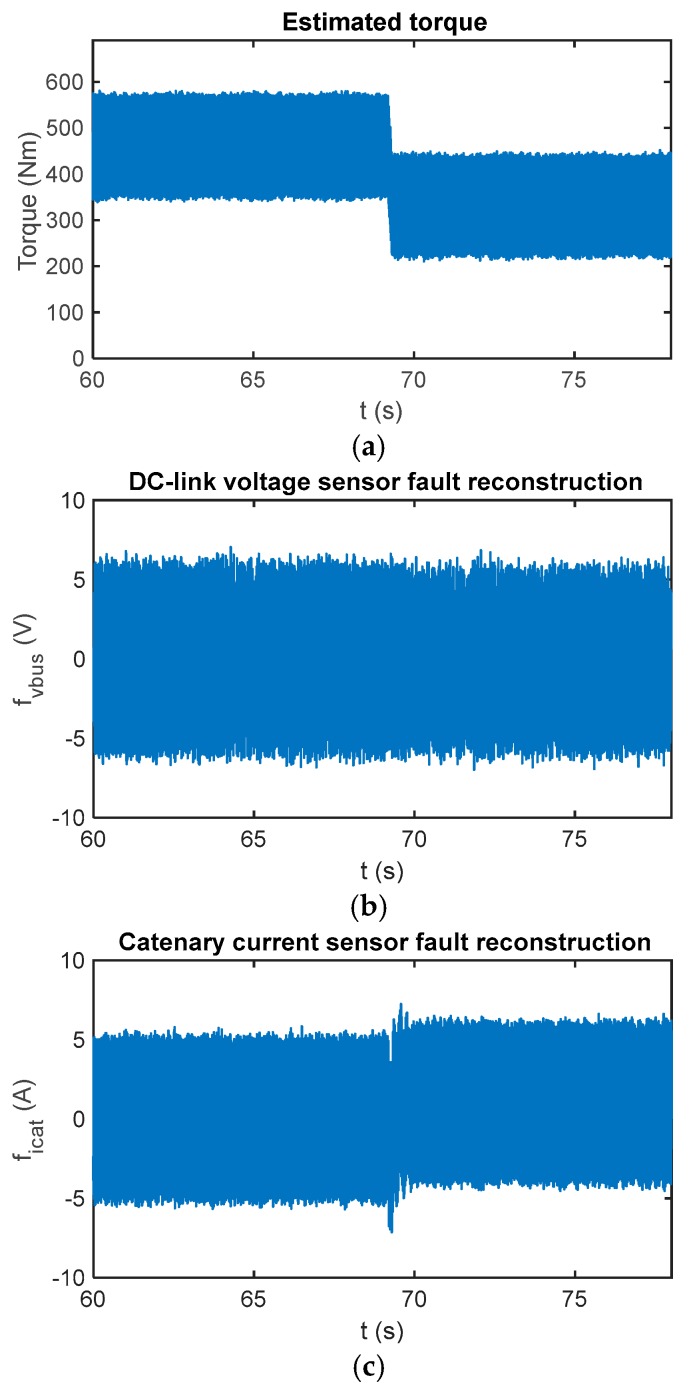
(**a**) Reference and estimated motor torque; (**b**) DC-link voltage sensor reconstruction in the case of a fault-free sensor in the HIL platform; and (**c**) catenary current sensor reconstruction in the case of a fault-free sensor in the HIL platform.

In Figure 14 the sensor fault reconstruction for a fault-free case is shown. Similar to simulations presented in the previous section, there is an oscillation in the fault reconstructions, the oscillation is due to the chattering effect. A first-order digital filter with a cut-off frequency equal to 5 Hz has been used to filter the reconstruction.

Different fault modes have been injected. Additive faults as offset faults, and multiplicative faults, the magnitude of which changes depending on the operating point, as gain faults. This magnitude change is especially evident for gain faults in the catenary current, as the catenary current value depends on the torque value.

The faults have been injected in different time instants in order to better show the effect and reconstruction of each one. The fault diagnosis method works right in the case of fault injections in the DC-link voltage and catenary current sensors at the same time instant, as well.

**Figure 15:**
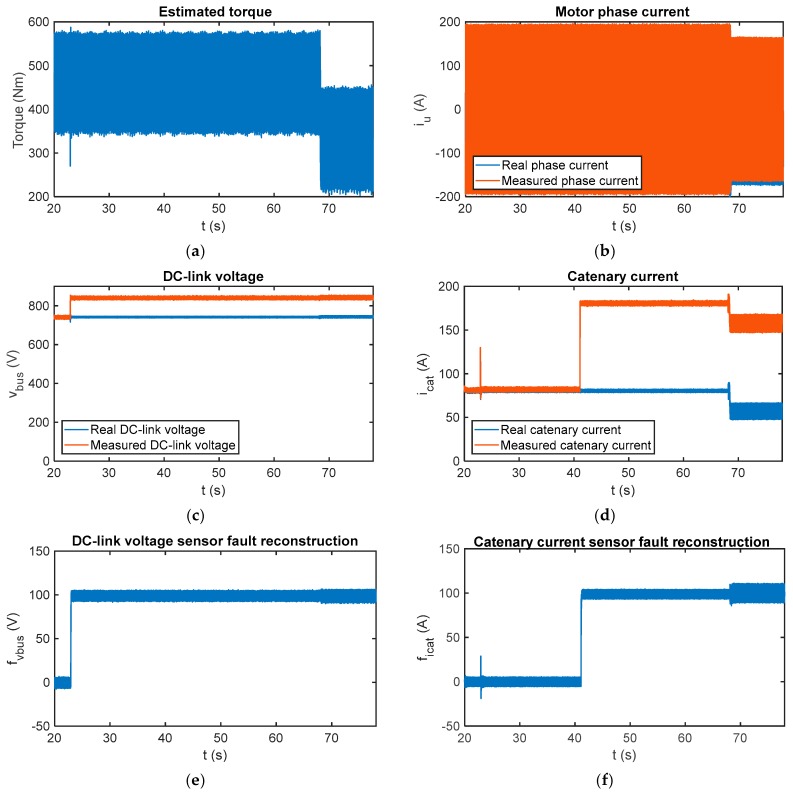
Fault reconstruction validation in the HIL platform for multiple injected faults. (**a**) Estimated motor torque; (**b**) measured and real phase current per motor; (**c**) measured and real DC-link; (**d**) measured and real catenary current; (**e**) DC-link voltage sensor fault reconstruction; and (**f**) catenary current sensor fault reconstruction.

In Figure 15, the fault reconstructions have been obtained for faulty DC-link voltage and catenary current sensors. A 100 V offset fault has been injected in the DC-link voltage sensor at *t* = 22.9 s and a 100 A offset fault in the catenary current sensor at *t* = 41.1 s. Moreover, a 20 A offset fault has been injected in the phase current sensor $i_{u}$ at *t* = 68.08 s. The DC-link voltage and catenary current sensor fault reconstructions have been correctly done, the average values for the period from *t* = 70 s to *t* = 78 s being 99.05 V and 99.73 A.

Furthermore, the offset fault injected in the phase current does not influence the average value, but increases the oscillation in the reconstruction, mainly in the catenary current fault reconstruction. This drawback has a limited effect as the final step of the presented fault detection algorithm calculates the average value of a number of samples for decision-making.

In conclusion, it is possible the sensor fault reconstruction although both sensors, DC-link voltage and catenary current, are faulty at the same time.

**Figure 16:**
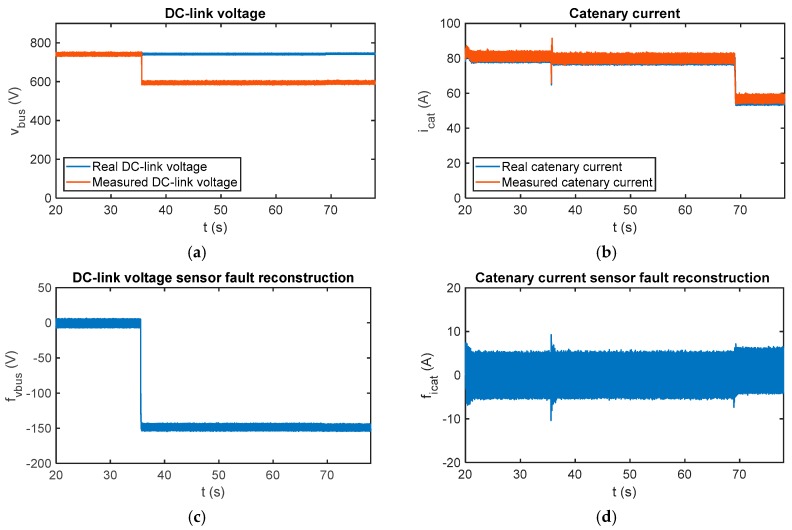
Fault reconstruction validation in the HIL platform for the gain fault injected in the DC-link voltage sensor. (**a**) Measured and real DC-link; (**b**) measured and real catenary current; (**c**) DC-link voltage sensor fault reconstruction; and (**d**) catenary current sensor fault reconstruction.

In case of multiplicative faults, the results for a gain fault injected in the DC-link voltage sensor are shown in Figure 16. From *t* = 35.8 s, the sensor measurement is 20% lower than the real value, so it decreases from 750 V to 600 V. The estimated motor torque is initially equal to 460 Nm and decreases to 323 Nm at *t* = 68.9 s. A comparison between real and measured values for DC-link voltage and catenary current is presented. A transient arises in the catenary current sensor fault reconstruction, when the DC-link sensor fault occurs, but it is not influenced in the steady state.

**Figure 17:**
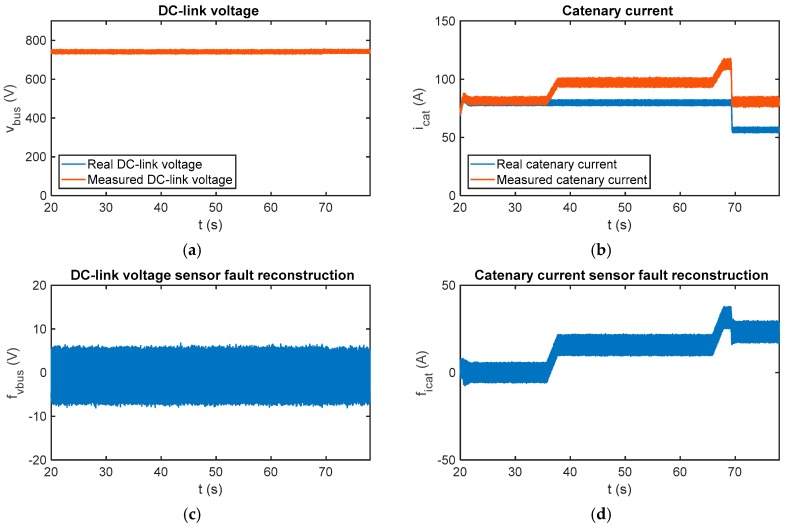
Fault reconstruction validation in the HIL platform for the gain fault injected into the catenary current sensor. (**a**) Measured and real DC-link; (**b**) measured and real catenary current; (**c**) DC-link voltage sensor fault reconstruction; and (**d**) catenary current sensor fault reconstruction.

In Figure 17 the sensor fault reconstructions for gain sensor faults injected in the catenary current measurement are presented. The measured catenary current is 20% over the real value from *t* = 35.75 s until and *t* = 65.9 s, and 40% over the real value from then on. It can be seen that the catenary current sensor fault reconstruction changes with the torque estimation, and this information is very useful to distinguish between offset and gain faults. The main difference between gain and offset faults is that the first ones are dependent on the operating point. Moreover, it can be see that the fault reconstruction for the DC-link voltage sensor is not influenced by the catenary current sensor faults. Both residuals are decoupled in the steady state, providing for easier logic for fault isolation.

## 6. Discussions

In this article, a sensor fault reconstruction for the DC-link voltage and catenary current sensors in a railway application has been presented. The solution proposed allows FDI and estimation of fault severity. The fault reconstruction is based on a SMO and equivalent injection signal. SMO has been proposed due to its robustness against uncertainties and disturbances. SMO-based fault estimation, under disturbances in the DC-link voltage measurement, is more robust than the Luenberger observer-based solution developed in a previous work [39].

Dynamic response of the fault reconstruction has been presented. As the railway traction drive is not a safety-critical system compared to aerospace systems, there is no need for instantaneous detection. Thus, decision-making is done after the reconstruction is over the threshold for some seconds in the steady state. Despite the lack of traction drive availability for some seconds, the train is able to keep operating, so the fault reconstruction is verified during some seconds in order to minimise the false detections. Effects of torque and catenary voltage changes on the fault reconstruction have been presented, too, showing a low impact in the steady state.

The sensitivity of fault reconstruction for input filter parameter changes has been presented, as well. A variation of ±100% has been analysed for series resistance and inductance. Similarly, DC-link capacitor changes have been simulated, with the exception of −100%, as this does not make any sense. The variation of the DC-link capacitor and series inductance do not have an impact in the steady state fault reconstruction, whereas the series resistor change impacts the DC-link voltage sensor fault reconstruction in the steady state. In the worst case, being that the resistor value is equal to twice the nominal one, the average voltage deviation is 7.88 V. Thresholds of ±15 V could be enough to avoid false detection in fault-free cases, due to parameter changes, taking into account the average voltage deviation and fault reconstruction oscillation.

The sensor fault reconstruction is implemented in a commercial TCU for a tram. The control strategy, safety, and diagnosis algorithms are running in the DSP of the TCU. The TCU is externally connected to a real-time simulator in a HIL platform, where the traction drive is modelled in MATLAB-Simulink. The fault reconstruction results are validated in the HIL platform.

An evaluation procedure is proposed for a railway application, where instantaneous fault diagnosis is not needed. Despite the decision-making being delayed for some seconds, it improves the robustness of the detection, reducing the false detections due to transients or disturbances. Due to reconstruction oscillations and a fault-free response, thresholds of ±20 V for the DC-link sensor and ±20 A for the catenary current sensor are recommended.

## 7. Conclusions

In this article, sensor fault reconstructions for DC-link voltage and catenary current sensors for a railway traction drive have been presented. Sensor fault reconstruction is based on a sliding mode observer and equivalent injection signal. The solution proposed shows robustness to parameter variations and noise in measurements. The solution proposed is able to detect multiple faults and provide the severity of the faults. The fault reconstruction algorithm has been implemented in a commercial traction unit control. Low computational cost and easiness to tune, for different traction unit configurations, are the main key points for industrial acceptance. A fault evaluation procedure for a railway application has been presented, as well. The fault reconstruction and evaluation can be adapted to electric drives in other applications.

## Figures and Tables

**Table 1:** Summary of sensors in the railway unit.

Sensor	Description
*v_cat_*	Catenary voltage sensors
*i_cat_*	Catenary current sensor
*i_ret_*	Return current to catenary sensor
*v_bus_*	DC-link voltage sensor
*i_crw_*	Braking unit current sensor
*i_u,v_*	Motor phase current sensors
*Encoder 1, 2*	Motor speed sensors

**Table 2:** Logic for DC-link voltage and catenary current sensor isolation.

Sensor Fault	Flag *i_cat_*	Flag *v_bus_*	Flag *v_cat_*	Flag *i_u,v_*
*i_cat_*	1	-	-	0
*v_bus_*	-	1	0	-
